# Uptake of evidence-based practice and its predictors among nurses in Ethiopia: a systematic review and meta-analysis

**DOI:** 10.3389/fphar.2024.1421690

**Published:** 2024-07-18

**Authors:** Muluken Amare Wudu, Setegn Mihret Tarekegn, Endalk Birrie Wondifraw, Tarikua Afework Birhanu, Molla Kassa Hailu, Yemane Eshetu Bekalu, Selamyhun Tadesse Yosef, Melaku Ashagrie Belete

**Affiliations:** ^1^ Department of Pediatrics and Child Health Nursing, College of Medicine and Health Sciences, Wollo University, Dessie, Ethiopia; ^2^ Department of Pediatrics and Child Health Nursing, School of Nursing and Midwifery, College of Medicine and Health Science, Wollo University, Dessie, Ethiopia; ^3^ Department of Public Health, ALKAN Health Sciences and Business College, Dessie, Ethiopia; ^4^ Department of Medical Laboratory Science, College of Medicine and Health Sciences, Woldiya University, Woldiya, Ethiopia; ^5^ Department of Medical Laboratory Science, College of Medicine and Health Sciences, Wollo University, Dessie, Ethiopia

**Keywords:** evidence based practice, uptakes, predictors, nurses, Ethiopia, systemic review, meta-analysis

## Abstract

**Background:** While evidence-based practice has demonstrated its capacity to enhance healthcare quality and bolster clinical outcomes, the translation of research into clinical practice encounters persistent challenges. In Ethiopia, there remains a dearth of comprehensive and nationally representative data concerning the extent of Evidence-based practice adoption among nurses. Thus, this systematic review and meta-analysis endeavors to assess the overall prevalence of Evidence-based practice implementation and delve into its determinants among Ethiopian nurses.

**Methods:** A systematic review and meta-analysis were conducted following the PRISMA guidelines. In order to identify pertinent studies, a search was conducted across PubMed, Scopus, Google Scholar, and EMBASE databases. A weighted inverse variance random-effects model was employed to estimate the pooled prevalence. Cochrane’s Q-test and I^2^ statistics were calculated to assess heterogeneity among studies. Funnel plots and Egger’s test were utilized to evaluate publication bias. Pooled implementation rates and meta-regression analysis were carried out using STATA 17.

**Results:** Of the total 1,590 retrieved articles, twelve studies including 4,933 nurses were included in the final analysis. The pooled prevalence of Evidence-based practice uptake among nurses in Ethiopia is 53% (95% CI: 46%–60%). Having knowledge about Evidence-based practice (AOR = 2.29; 95% CI: 1.90, 2.69; I^2^ = 70.95%), holding a favorable attitude towards Evidence-based practice (AOR = 2.56; 95% CI: 1.63, 3.49; I^2^ = 88.39%), occupying a head nurse position (AOR = 3.15; 95% CI: 1.85, 4.46; I^2^ = 87.42%), possessing effective communication skills (AOR = 4.99; 95% CI: 1.47, 8.51; I^2^ = 99.86%), and having access to Evidence-based practice guidelines (AOR = 1.90; 95% CI: 1.55, 2.24; I^2^ = 57.24%) were identified as predictors of the uptake of Evidence-based practice.

**Conclusion:** Only half of Ethiopia’s nurses exhibit a strong embrace of Evidence-Based Practice within clinical settings, underscoring the urgent necessity for coordinated endeavors to cultivate this essential practice. Possessing knowledge, effective communication skills, access to updated guidelines, maintaining a positive attitude towards Evidence-Based Practice, and holding a position as head nurse emerged as predictors of successful implementation of Evidence-Based Practice. Hence, policymakers must prioritize capacity-building initiatives, disseminate the latest EBP guidelines widely, and strengthen mentorship roles for head nurses.

**Systematic Review Registration:** https://www.crd.york.ac.uk/prospero/#searchadvanced, identifier CRD42023488943

## Introduction

Evidence-based practice (EBP) is an approach to problem-solving that integrates the latest information, the knowledge of care providers, and the preferences and values of patients (Leen et al., 2014; Melnyk and Fineout-Overholt, 2022). It entails making informed decisions using the most reliable available evidence from various sources in a deliberate, clear, and rational manner (Melnyk and Fineout-Overholt, 2022).

EBP comprises five essential steps: formulating patient-centered questions, obtaining the latest evidence, critically evaluating the evidence for its reliability and relevance, applying the evidence through shared decision-making, and ultimately assessing the outcomes while making adjustments to practice as necessary (Leen et al., 2014).

Nursing serves as the foundation of the global healthcare structure, and nursing EBP is essential for delivering excellent patient care (World health organization WHO, 2017). Consequently, research and practice coexist in close proximity (Kristensen et al., 2015). While EBP is employed across various professions as a method of professional practice, its growth in the field of nursing is rapid (Jordan et al., 2016).

EBP has a favorable impact on nurses’ practices, enabling them to transition from outdated methods to a scientifically-based approach (Oluwatoyin and EkeFJH, 2016). Furthermore, it leads to reduced costs, improved patient outcomes, and establishes a foundation for high-quality patient care (Branham et al., 2014; Melnyk and Fineout-Overholt, 2022). Additionally, it enhances satisfaction among patients and their families and contributes to career advancement (Jordan et al., 2016). Moreover, it significantly enhances job satisfaction (WHO, 2015), boosts productivity, reduces overtime (Jane Vortherms et al., 2015), and bridges the gap between theory and application (Saleh, 2018).

Despite the growing accessibility of healthcare data and consistent government oversight, the implementation of evidence-based practice in the nursing profession remains limited (Melnyk and Fineout-Overholt, 2022). In developing countries, the utilization of EBP is uncommon, and for many healthcare organizations, engaging in EBP processes represents new and often daunting responsibilities (Williams et al., 2015; Labrague et al., 2019).

Evidence-based preventive interventions have the potential to address over half of the 2.5 million deaths that occur globally each year (World health organization WHO, 2017). However, the underutilization of evidence-based practice (EBP) in decision-making often leads to avoidable deaths, diminished quality of life, and unfavorable outcomes for patients (Promoting evidence-based health care in Africa, 2017).

The literature highlights that subpar evidence-based practice (EBP) among nurses is linked to several factors, including insufficient nursing skills and knowledge, limited access to information, time constraints during work hours, a scarcity of training and resources, heavy workloads, and a lack of supportive peers. These challenges pose significant obstacles for nurses serving vulnerable populations in low-income countries like Ethiopia (Hadgu et al., 2015; Hoyiso et al., 2018; Tadesse et al., 2018; Dereje et al., 2019; Alemayehu and Jevoor, 2021; Alene et al., 2021; Assefa and Shewangizaw, 2021; Aynalem et al., 2021; Dagne et al., 2021; Degu et al., 2022; Lamesa et al., 2023; Megersa et al., 2023).

Despite Ethiopia’s commitment to enhancing evidence-based clinical decision-making through the signing of the Kigali Agreement (CEBHA, 2012) and the provision of EBP training for nurses (CEBHA, 2014), the implementation of EBP and its determinants have been inconsistently documented across the country. Furthermore, despite the significant amount of time nursing professionals spend with patients, there is a lack of systematic review and meta-analysis studies on EBP in Ethiopia. In order to address discrepancies between findings and enhance the quality of patient care, it is crucial to identify the most effective evidence-based interventions. This study aims to address this gap by providing baseline information on the implementation of EBP among nurses nationwide, along with recommendations regarding its determinants. Consequently, it has the potential to catalyze action among health and nurse managers, as well as policymakers, to strengthen the integration of EBP into nursing care, thereby ensuring the delivery of high-quality care.

## Methods and materials

### Protocol registrations

The search initially focused on narrative analysis and systematic reviews, along with registered protocols, to prevent duplications. Subsequently, the protocol for this systematic review and meta-analysis was registered in the International Prospective Register of Systematic Reviews (PROSPERO) (https://www.crd.york.ac.uk/prospero/display_record.php?ID=CRD42023488943). Furthermore, the methodology for conducting the systematic review and meta-analysis was established in accordance with the PRISMA (Preferred Reporting Items for Systematic Reviews and Meta-Analyses) reporting checklist (Supplementary Material S1) (Page et al., 2021).

### Inclusion and exclusion criteria

The inclusion criteria for this study comprised observational studies (including cross-sectional, retrospective cohort, and case-control studies) conducted in public hospitals in Ethiopia. These studies needed to be published articles or grey literature reports written in English since 2012. Additionally, only primary studies assessed with moderate to low risk of bias according to the Joanna Briggs Institute (JBI) criteria were considered. On the flip side, it's important to note that this study specifically excluded research conducted outside of Ethiopia, observational studies not presented in English, purely qualitative research, case studies, case reports, and randomized trials.

### Information source

A thorough search was carried out across various online databases, including PubMed, Scopus, EMBASE, and other sources of gray literature, such as Google Scholar, dissertations, and governmental or private sector reports, up until 7 February 2024.

### Searching strategy

We considered all studies involving human subjects conducted prior to the search date. Our approach involved conducting sensitive searches that combined text words using Boolean operators, all guided by the **CoCo Pop** mnemonic principle. The criteria were as follows: **Co**ndition—uptake of evidence-based practice; **Co**ntext—Ethiopia; **Pop**ulation—nurses working in hospitals. Accordingly, using the phrase “Uptake of Evidence-Based Practice and Its Predictors among Nurses in Ethiopia,” we screened some studies from gray literature. A Medical Subject Headings (MeSH) thesaurus and keyword terms and phrases were utilized both independently and in combination using the boolean operators “OR” and “AND” to search for eligible articles. The authors implemented the following search strategy: (((((((((Evidence-based practice) AND (implementation)) OR (uptake)) OR (utilization)) AND (associated factors)) OR (predictors)) OR (determinants)) AND (among nurse*)) AND (in Ethiopia). Furthermore, a cross-reference search was conducted to identify additional related studies that might not have been captured by the initial database search, among the final included studies (Supplementary Material S2).

### Study selection process

Observational studies (including cross-sectional, retrospective cohort, and case-control studies) conducted at the national, regional, or district levels of health facilities among nurses regarding the uptake of evidence-based practice and its predictors were included. Subsequently, four authors (MA, SM, MA, and EB) independently screened the titles and abstracts of the studies. Following this, the full texts were scrutinized based on the eligibility criteria, and duplicate articles were managed using EndNote 20 citation manager. Any discrepancies were resolved through open discussion with the remaining three authors (TA, ST, YE, and MK).

### Data extraction

Four authors (MA, SM, FA, and EB) meticulously extracted the data in an organized manner using a Microsoft Excel spreadsheet. Any disagreements between authors were resolved through open conversation involving the remaining three authors (TA, ST, and MK). Moreover, we attempted to communicate via email with the corresponding authors of non-open-access works. Papers were excluded from the final analysis if the authors were unable to provide the complete text. The data extraction encompassed various elements, including the last name of the first author, publication year, study regions, study settings, hospital levels, study design, types of data instruments, sample size, response rate, proportion of EBP implementations, effect size, predictors with a 95% confidence interval, and standard error of proportion and odds ratio.

### Outcomes

The primary outcome of the study was to determine the pooled prevalence of EBP implementation and its predictors within hospital settings in Ethiopia. To achieve this, adjusted odds ratios, standard errors, and pooled effect sizes with 95% confidence intervals (CI) were calculated to identify the factors contributing to the good uptake of EBP.

### Study risk of bias assessment

The risk of bias for the studies included in the review was assessed independently by the four authors (MA, SM, FA, and EB) using the Joanna Briggs Institute (JBI) critical appraisal checklists. Bias assessment was based on several criteria, including sample inclusion criteria, descriptions of research subjects and settings, measurement validity and reliability, handling of confounding factors, and appropriateness of outcome measures. Studies scoring 7 or higher were classified as low risk, those scoring 5–6 as medium risk, and those scoring 4 or less as high risk. The review was then extended to include trials with low and medium risk. Any discrepancies were resolved through discussion, and when necessary, a third reviewer was consulted (Table 1).

**TABLE 1:** Quality assessment for the included Studies in Ethiopia, 2024 (N = 12).

Studies	The criteria for inclusion in the sample clearly defined	Describe study setting and participant	Valid and reliable exposure measurement	Objective and standard criteria for measurement	Identified confounder	Strategies to deal with confounders	Valid and reliable outcome measurement	Appropriate statically analysis	Frequency and percent of “yes” (%)	Judgement/risk of bias
Megersa et al. (2023)	Yes	Yes	Yes	Yes	Yes	Yes	Yes	Yes	8/8 = 100	Low risk
Aynalem et al. (2021)	Yes	Yes	Yes	Yes	Yes	Yes	Yes	Yes	8/8/ = 100	Low risk
Degu et al. (2022)	Yes	Yes	Yes	Yes	Yes	Yes	Yes	Yes	8/8 = 100	Low risk
Dereje et al. (2019)	Yes	No	Yes	Yes	Yes	Yes	Yes	Yes	7/8 = 87.5	Low risk
Hoyiso et al. (2018)	No	No	Yes	Yes	Yes	Yes	Yes	Yes	6/8 = 75	Moderate risk
Alemayehu and Jevoor (2021)	Yes	Yes	Yes	Yes	Yes	Yes	Yes	Yes	8/8 = 100	Low risk
Alene et al. (2021)	No	Yes	Yes	Yes	Yes	Yes	Yes	Yes	7/8 = 87.5	Low risk
Assefa and Shewangizaw (2021)	No	Yes	Yes	Yes	Yes	Yes	No	Yes	6/8 = 75	Moderate risk
Tadesse et al. (2018)	No	Yes	Yes	Yes	Yes	Yes	No	Yes	6/8 = 75	Moderate risk
Dagne et al. (2021)	Yes	yes	Yes	Yes	Yes	Yes	Yes	Yes	8/8 = 100	Low risk
Hadgu et al. (2015)	Yes	Yes	Yes	No	Yes	Yes	Yes	Yes	6/8 = 75	Moderate risk
Lamesa et al. (2023)	Yes	Yes	Yes	Yes	Yes	Yes	Yes	Yes	8/8 = 100	Low risk

To determine the presence of publication bias, we employed a combination of graphical analyses, specifically funnel plots, and objective statistical test such as Egger’s tests. These methodologies were utilized to comprehensively evaluate the potential impact of publication bias, particularly the influence of small studies on the overall results. Consequently, the findings exhibited symmetry and demonstrated a *p*-value > 0.05, indicating the absence of a small study effect in this study. Furthermore, the Doi plot was employed to discern bias and assess the certainty of evidence, revealing a Luis Furuya Kanamori (LFK) index of 0.16. This value suggests the absence of asymmetry, indicating that no small study effect was observed in the overall results.

### Data synthesis and analysis

The pooled prevalence of evidence-based practice (EBP) uptake and its predictors were analyzed using STATA Version 17 software. The pooled estimate was calculated employing the random-effects Der Simonian-Laird model method. To assess the heterogeneity of the studies, the Cochrane Q-test and I^2^ statistics were utilized. Notably, significant heterogeneity was observed across the studies (I^2^ = 95.99%, *p*-value<0.001). In response to this heterogeneity, a subgroup analysis was conducted based on various factors including publication year, study design, sample size, study regions, instrument type, and levels of hospitals. Despite these efforts, the heterogeneity persisted. Hence, a thorough assessment of publication bias and sensitivity analysis was conducted, revealing no indication of small-study effects nor any single study significantly influencing the aggregated adoption of EBP and its determining factors in Ethiopia.

## Result

**FIGURE 1:**
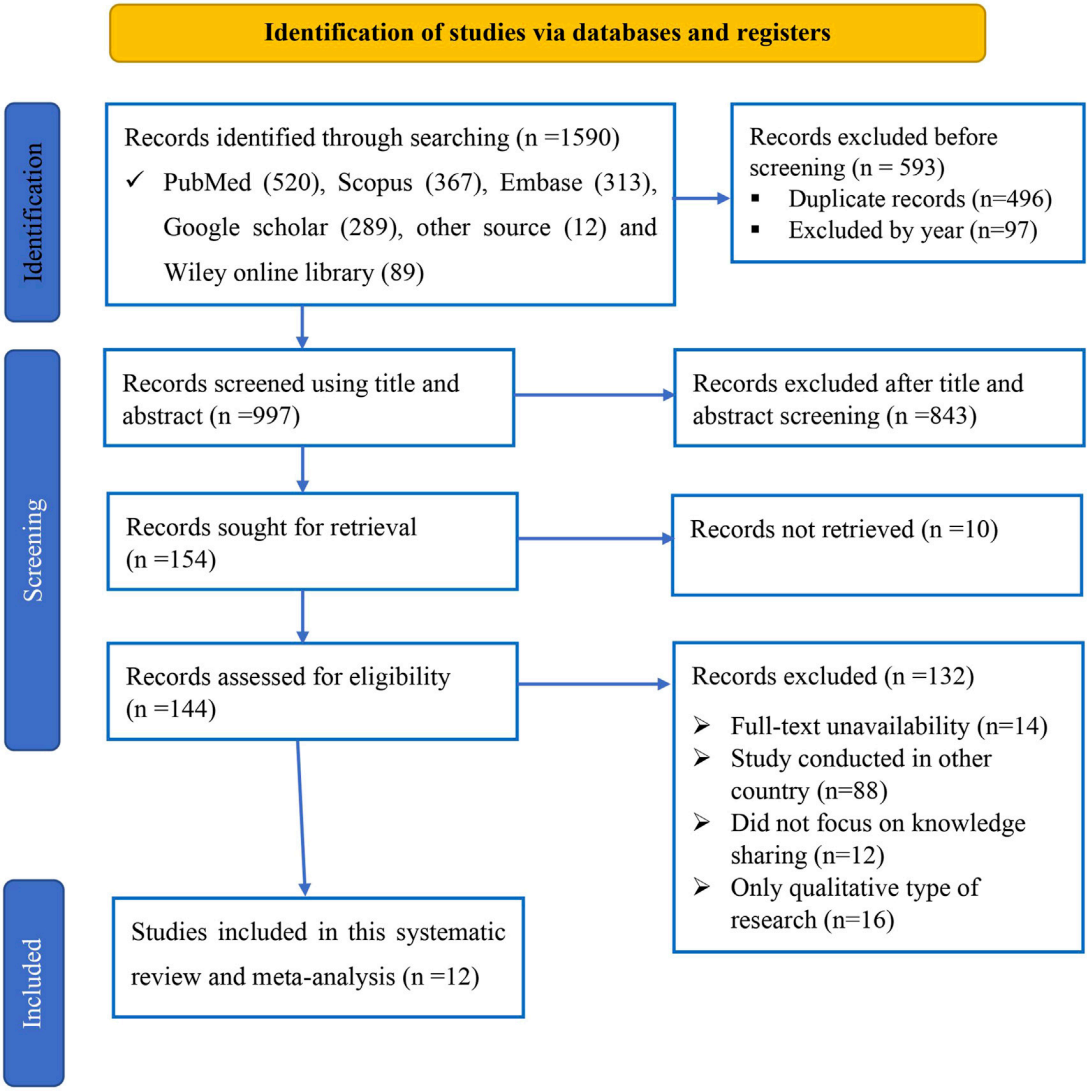
Study selection for systemic review and meta-analysis of evidence-based practice uptake and associated factors among nurses in Ethiopia, 2024.

A total of 1,590 articles were initially gathered, with 593 records excluded prior to screening. Subsequently, 843 articles were further excluded after a thorough examination of their titles and abstracts. Additionally, 10 records could not be retrieved for evaluation. After a meticulous assessment of eligibility criteria, 132 records were deemed unsuitable and excluded. Finally, 12 studies met the inclusion criteria and were selected for systematic review and meta-analysis (Figure 1).

### Characteristics of the included studies

In this systematic review, a total of 12 studies were included. Out of the overall participant pool of 5,097 individuals, 4,933 actively engaged in the data collection process, leading to a remarkable response rate of 96.78%. These studies were distributed across various regions, with 4 from Addis Ababa, 3 from Amhara, 3 from Oromia, and the remaining 2 from SNNPS region. Furthermore, among the 12 studies, 5 were published before 2020, while the remaining 7 were published in 2020 or later. In terms of hospital levels, six studies encompassed all hospital levels, whereas the other six were conducted exclusively in higher-level hospitals. Additionally, 7 studies adopted a cross-sectional study design, while 5 utilized a mixed-type design. Regarding the type of data instruments utilized, 7 studies adapted the Evidence-Based Practice Questionnaire (EBPQ) in conjunction with the Barriers’ Scale. Additionally, 2 studies employed a combination of the EBPQ and the Critically Appraised Topics (CAT). Furthermore, one study utilized Funk’s Barriers scale, another employed a combination of the EBPQ and EBP-COQ, and the remaining study utilized the EBP-COQ alongside the Barriers’ Scale (Table 2).

**TABLE 2:** Characteristics of individual studies in Ethiopia, 2024 (N = 12).

Authors and publication year	Regions	Study setting	Level of hospital	Study design	Instrument	Sample size	Response	Event	Outcome	Prevalence	Quality assessment
Megersa et al. (2023)	Oromia	Hospital-based	Primary, General and Referral hospitals	Cross-sectional	EBPQ and Barriers’ Scale	418	403	211	EBP implementation	0.524	Low risk
Aynalem et al. (2021)	Amhara	Hospital-based	Referral hospital	Cross-sectional	EBPQ and CAT	684	671	369	EBP implementation	0.55	Low risk
Degu et al. (2022)	Amhara	Hospital-based	Teaching hospital	Mixed	EBPQ and Barriers’ Scale	530	507	239	EBP implementation	0.471	Low risk
Dereje et al. (2019)	Oromia	Hospital-based	Teaching & General hospitals	Mixed	EBP-COQ and BARRUERS Scale	270	253	131	EBP implementation	0.517	Moderate risk
Hoyiso et al. (2018)	Oromia	Hospital-based	Teaching and General hospital	Mixed	Funk’s BARRIERS scale	265	245	97	EBP implementation	0.395	Low risk
Alemayehu and Jevoor (2021)	SNNPR	Hospital-based	Referral hospital	Cross-sectional	EBPQ and EBP-COQ	684	671	369	EBP implementation	0.55	Low risk
Alene et al. (2021)	Addis Ababa	Hospital-based	Referral hospital	Cross-sectional	EBPQ and Barriers’ Scale	140	135	115	EBP implementation	0.85	Low risk
Assefa and Shewangizaw (2021)	Addis Ababa	Hospital-based	Referral and General	Mixed	EBPQ and Barriers’ Scale	422	422	243	EBP implementation	0.575	Moderate risk
Tadesse et al. (2018)	SNNPR	Hospital-based	Teaching hospital, comprehensive, General hospitals	Cross-sectional	EBPQ and Barriers’ Scale	223	208	80	EBP implementation	0.384	Moderate risk
Dagne et al. (2021)	Amhara	Hospital-based	Teaching, General, primary hospitals	Cross-sectional	EBPQ and Barriers’ Scale	826	790	274	EBP implementation	0.346	Low risk
Hadgu et al., (2015)	Addis Ababa	Hospital-based	Teaching hospital	Mixed	EBPQ and Barriers’ Scale	217	210	121	EBP implementation	0.576	Moderate risk
Lamesa et al. (2023)	Addis Ababa	Hospital-based	Teaching hospital	Cross-sectional	EBPQ and CAT	418	418	243	EBP implementation	0.581	Low risk
Total						5,097	4,933	2,492		0.53	

### The pooled uptake of EBP in Ethiopia

**FIGURE 2:**
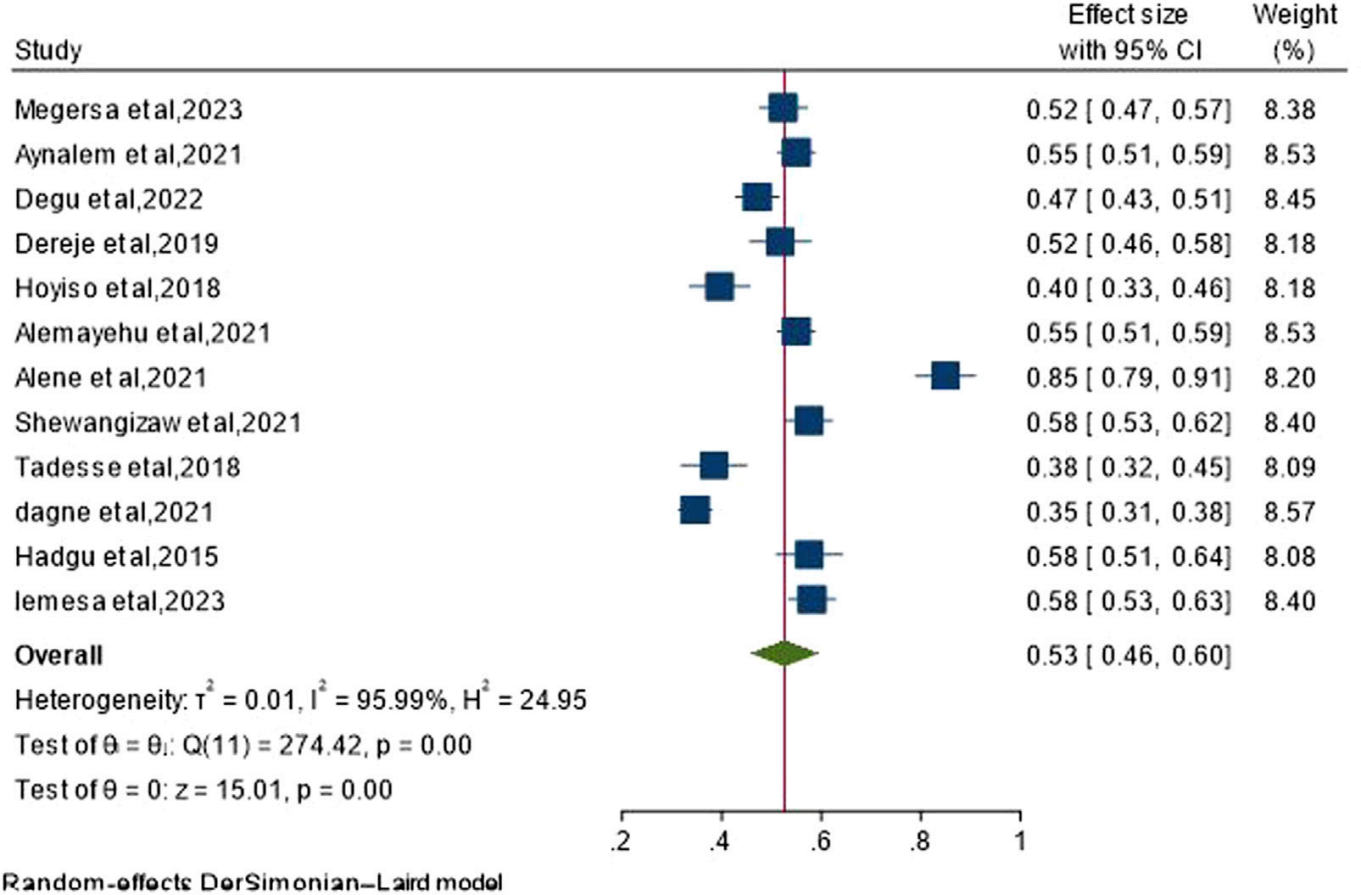
Pooled magnitude of uptake of EBP among nurses in Ethiopia, 2024.

Out of the total 12 studies incorporated into this meta-analysis and systematic review utilizing a random-effect model, the overall pooled prevalence of EBP uptake among nurses in Ethiopia is 53% (95% CI: 46%–60%), revealing significant heterogeneity across studies (I^2^ = 95.99%, *p*-value < 0.001) (Figure 2).

### Sub-group analysis for EBP uptake

Subgroup analyses were performed to tackle the elevated levels of heterogeneity observed in the included studies, which were apparent both subjectively through tools like the forest plot, and quantitatively through metrics such as I^2^ and the Q test. A subgroup analysis was undertaken, considering various factors such as publication year, study design, sample size, study regions, instrument type, and levels of hospitals. However, despite these efforts, the heterogeneity persisted (Table 3).

**TABLE 3:** Subgroup analysis of EBP implementation studies among nurses in Ethiopia, 2024.

Sub-groups		Number of studies	Effect size (Random, 95% CI)	Heterogeneity (I^2^)	*p*-value
Sample size	<384	5	0.55 (0.38, 0.72)	97.25%	<0.001
	≥384	7	0.51 (0.44, 0.58)	94.97%	<0.001
	Overall	12	0.53 (0.46,0.60)	95.99%	<0.001
Regions	Addis Ababa	4	0.65 (0.52,0.77)	95.23%	<0.000
	Amhara	3	0.46 (0.33, 0.58)	96.92%	<0.000
	Oromia	3	0.48 (0.40,0.58)	82.78%	<0.000
	SNNPS	2	0.47 (0.31,0.63)	94.49%	<0.000
	Overall	12	0.53 (0.46,0.60)	95.99%	<0.000
Instrument type	EBPQ and Barriers’ Scale	7	0.53 (0.41,0.65)	97.5%	<0.000
	EBPQ and CAT	2	0.56 (0.53,0.59)	3.16%	<0.31
	Overall	12	0.53 (0.46,0.60)	95.99%	<0.000
Publication year	<2020	5	0.48 (0.41,0.55)	85.52%	<0.000
	≥2020	7	0.56 (0.46, 0.66)	97.51%	<0.000
	Overall	12	0.53 (0.46, 0.60)	95.99%	<0.000
Study design	Mixed	5	0.51 (0.44,0.57)	85.53%	<0.000
	Cross-sectional	7	0.54 (0.43,0.65)	97.57%	<0.000
	Overall	12	0.53 (0.46,0.60)	95.99%	<0.000
Levels of hospitals	All levels	6	0.46 (0.37,0.54)	94.06%	<0.000
	Higher level	6	0.59 (0.51, 0.68)	95.25%	<0.000
	Overall	12	0.53 (0.46,0.60)	95.99%	<0.000

### Publication bias assessment

**FIGURE 3:**
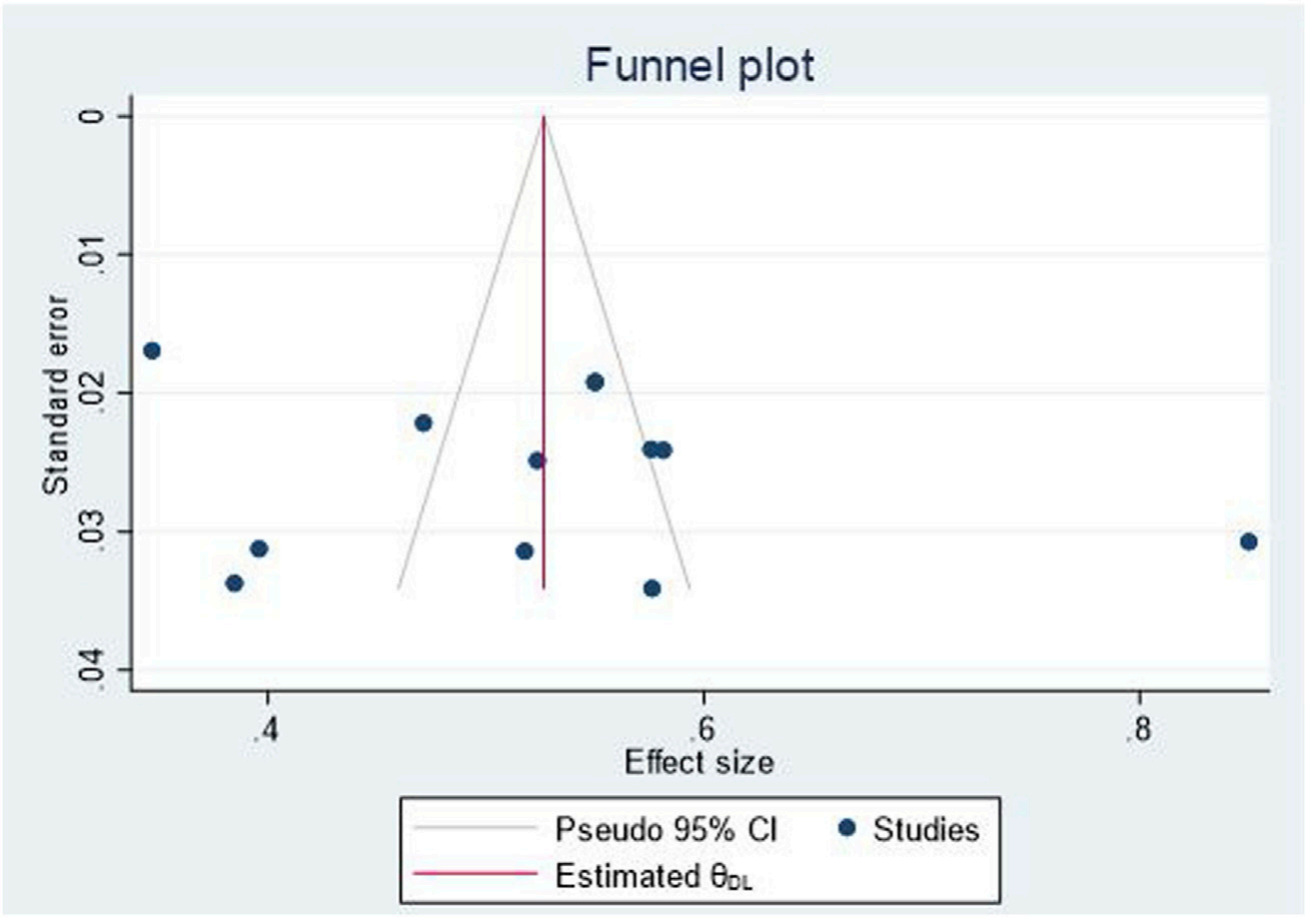
Egger test for analysis of publication bias for EBP uptake among nurses in Ethiopia, 2024.

**FIGURE 4:**
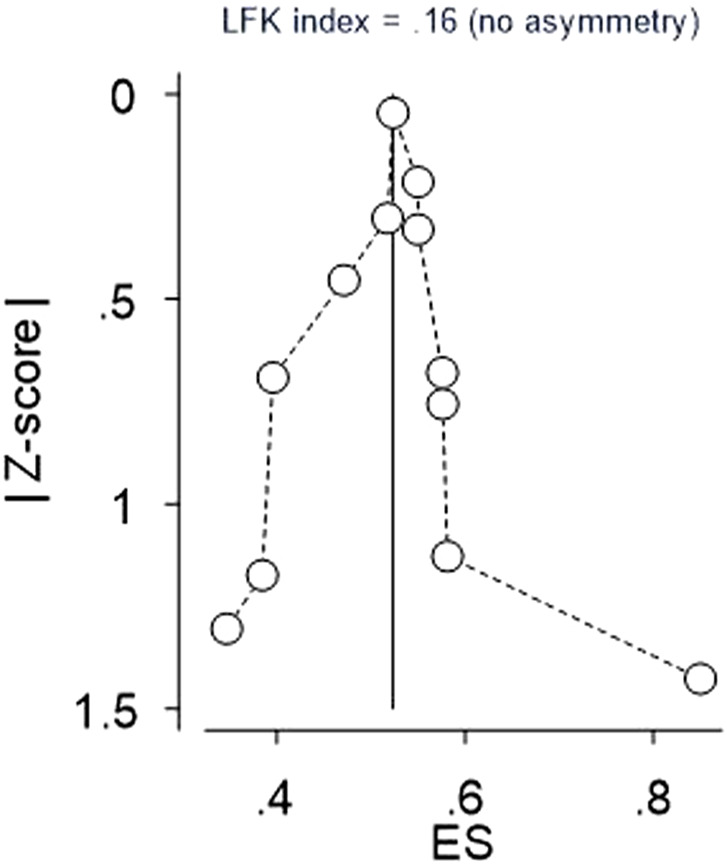
Doi plot for Reporting bias and certainty of evidence for EBP uptake among nurses in Ethiopia, 2024.

To assess the potential presence of publication bias, funnel plots and objective tests such as Egger’s statistical tests and Doi plot were employed. Visual inspection of the funnel plot revealed slight dispersion of studies (outliers), particularly on the right side (see figure). Subsequently, Egger’s test was conducted, yielding a non-significant *p*-value of 0.541, indicating the absence of small study effects on the overall study result (Figure 3). Similarly, the Doi plot was utilized to identify bias and evaluate the certainty of evidence, resulting in a Luis Furuya Kanamori (LFK) index of 0.16. This value suggests the absence of asymmetry, indicating that no small study effect was observed in the overall results (Figure 4).

### Sensitivity analysis

**FIGURE 5:**
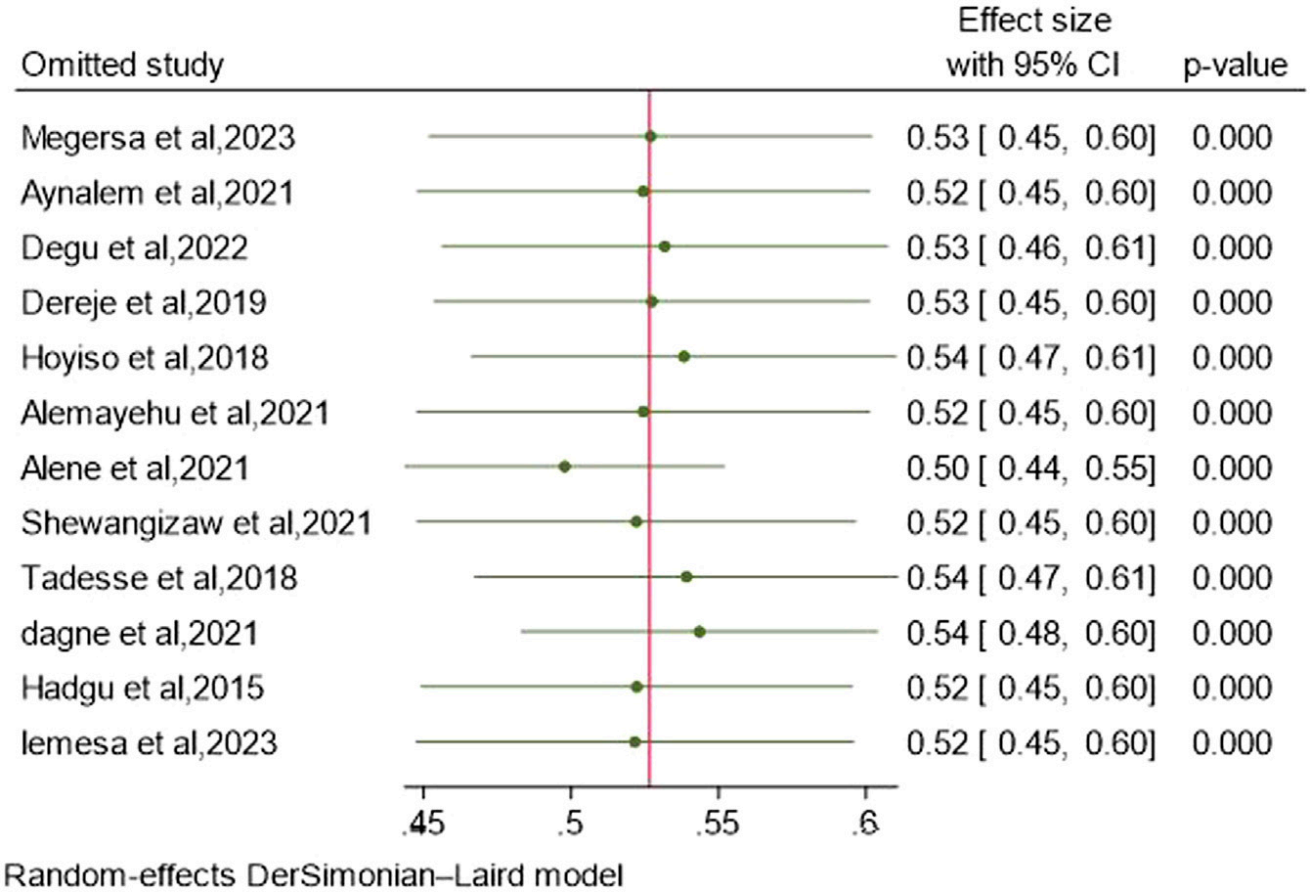
Sensitivity analysis for EBP implementation among nurses in Ethiopia, 2024.

To evaluate the influence of a particular study on the overall meta-analysis outcome and to address any potential outliers, sensitivity analyses or influential analyses were conducted. The results indicate that the inclusion or exclusion of outlier studies did not substantially affect the pooled EBP uptake, as the point estimate of the individual study fell within the confidence interval of the combined analysis, suggesting that the heterogeneity (as measured by I^2^) prior to sensitivity analysis had no impact on the overall effect size (Figure 5).

### Predictors of uptake of EBP

In this review, factors such as familiarity with EBP, a positive attitude towards its implementation, holding a head nurse position, possessing effective communication skills, and the availability of EBP guidelines were identified as predictors of EBP adoption among nurses. However, six studies indicated that the level of education, as well as factors such as time and internet access, were not associated with the uptake of EBP.

Six studies (Hadgu et al., 2015; Dereje et al., 2019; Alemayehu and Jevoor, 2021; Aynalem et al., 2021; Dagne et al., 2021; Megersa et al., 2023) revealed that nurses with a strong grasp of EBP were twice as likely to implement it in their setting compared to their counterparts [AOR = 2.29; 95% CI: (1.90, 2.69); I2 = 70.95%]. Similarly, three studies (Aynalem et al., 2021; Dagne et al., 2021; Degu et al., 2022) were included to evaluate the relationship between attitude and EBP uptake among nurses. It was found that nurses with a favorable attitude towards EBP were 2.56 times more likely to implement EBP in their setting compared to their counterparts [AOR = 2.56; 95% CI: (1.63, 3.49); I^2^ = 88.39%]. Furthermore, four studies (Tadesse et al., 2018; Dereje et al., 2019; Dagne et al., 2021; Megersa et al., 2023) revealed that nurses holding a head nurse position were 3.15 times more likely to implement EBP in their setting compared to ordinary nurses [AOR = 3.15; 95% CI: (1.85, 4.46); I^2^ = 87.42%]. Four studies (Hadgu et al., 2015; Alemayehu and Jevoor, 2021; Aynalem et al., 2021; Dagne et al., 2021) suggested that nurses with effective communication skills were nearly five times more likely to implement EBP in their setting compared to their counterparts [AOR = 4.99; 95% CI: (1.47, 8.51); I^2^ = 99.86%]. Finally, four included studies (Alemayehu and Jevoor, 2021; Aynalem et al., 2021; Dagne et al., 2021; Degu et al., 2022) suggested that nurses with access to EBP guidelines in their setting were nearly two times more likely to implement EBP compared to their counterparts [AOR = 1.90; 95% CI: (1.55, 2.24); I2 = 57.24%] (Table 4).

**TABLE 4:** Meta regression analysis (predictors) of good uptake of EBP among nurses in Ethiopia, 2024.

Factors	Number of studies	Odds ratio (Random, with 95% CI)	Heterogeneity (I^2^)(%)	*p*-value
Knowledgeable about EBP	6	2.29 (1.90, 2.69)	70.95	0.000
Had favorable attitude	3	2.56 (1.63, 3.49)	88.39	0.000
Head nurse	4	3.15 (1.85, 4.46)	87.42	0.000
Had effective communication	4	4.99 (1.47, 8.51)	99.86	0.000
Availability of EBP guideline	4	1.90 (1.55, 2.24)	57.24	0.000
Level of educations	3	1.75 (0.35, 3.16)	91.82	0.000
Time and internet access	3	3.71 (0.88, 6.54)	98.99	0.000

## Discussion

Evidence-based practice (EBP) yields numerous advantages for the nursing profession, encompassing heightened job satisfaction, empowerment, enhanced skills for incorporating patient preferences into practice, support for professional advancement, and ongoing career development through specialized roles (Burman et al., 2013; Melnyk et al., 2018; Melnyk and Fineout-Overholt, 2022). Furthermore, EBP exerts a positive influence on the healthcare system, manifesting in improved care quality, superior patient outcomes, heightened patient safety, cost reduction, fortified foundations for healthcare investment decisions, and capacity enhancement through collaborative efforts (WHO, 2015; Kim et al., 2016). Therefore, this systematic review and meta-analysis were conducted to determine the pooled extent of evidence-based practice adoption and its predictors among Ethiopian nurses. The findings revealed that half of the nurses demonstrated proficient implementation of evidence-based practice, indicating a need for further concerted efforts in this domain.

In the present study, the collective estimate of evidence-based practice (EBP) uptake among nurses in Ethiopia stood at 53% (95% CI: 46%–60%). This discovery resonates with previous research conducted within Ethiopia (Wubante and Tegegne, 2022; Zewdie et al., 2023). Furthermore, our findings align with those of a global systematic review and meta-analysis (Li et al., 2019). However, it’s worth noting that our study’s aggregated estimate of EBP implementation falls below that reported in studies from China and Sweden (Brämberg et al., 2017; Zhao et al., 2022). The variation observed could stem from several factors, including the level of healthcare setting, the influence of EBP implementation on nurses’ career development systems, disparities in nurses’ knowledge and skill levels regarding EBP, as well as variances in the supportive systems within healthcare organizations (World health organization WHO, 2017).

Nurses who demonstrated a strong understanding of EBP were twice as likely to implement it within their respective settings, as revealed by the findings of the current study. This finding corroborates similar results observed in studies conducted in Ethiopia (Wubante and Tegegne, 2022; Zewdie et al., 2023); global (Li et al., 2019); China (Zhao et al., 2022); Sweden (Brämberg et al., 2017); low- and middle-income country (Shayan et al., 2019); worldwide review (Furtado et al., 2024) contexts; Cyprus (Pitsillidou et al., 2020); Pakistan (Aslam et al., 2023); among developed countries (Riahi and Khajehei, 2019); Switzerland (Verloo et al., 2017); and the USA (44,45). This phenomenon may be attributed to the fact that possessing strong knowledge of EBP not only boosts the confidence of healthcare professionals but also enhances their skills to effectively implement it. This suggests that the consistency of EBP implementation is correlated with knowledge. Consequently, nursing leaders and associations must actively promote continuous career development to enhance EBP knowledge, The healthcare system and its managers should actively promote nurses’ knowledge of EBP through capacity-building approaches such as training sessions and the dissemination of updated guidelines. Encouraging nurses to engage in high-quality research and integrating EBP into educational curricula will also enhance their understanding of EBP. These efforts will ultimately lead to increased implementation rates of EBP.

The meta-regression results of the current study indicated that nurses who held favorable attitudes towards EBP were more inclined to implement it compared to those with unfavorable attitudes. This finding is consistent with similar results observed in Ethiopia (Wubante and Tegegne, 2022; Zewdie et al., 2023), as well as in global (Li et al., 2019), China (Zhao et al., 2022), low- and middle-income country (Shayan et al., 2019), and worldwide review (Furtado et al., 2024) contexts; among developed countries (Riahi and Khajehei, 2019), the USA (Godshall, 2016; Stavor et al., 2017), Saudi Arabia (Alqahtani et al., 2020), and Norway (Stokke et al., 2014). This could be explained by EBP enhancing nurses’ capacity to evaluate and incorporate information for clinical decisions, resulting in favorable outcomes. Further endeavors should focus on increasing professionals’ understanding of the advantages of EBP in improving clinical care and patient outcomes, thereby fostering better practice. This suggests that further efforts are required to cultivate nurses’ favorable attitudes towards EBP implementation. Consequently, nurse managers should emphasize recognizing nurses for better patient outcomes and integrating EBP into career development as a performance indicator. This approach will enhance nurses’ attitudes towards EBP implementation.

Nurses holding a head nurse position were three times more likely to implement EBP in their setting compared to ordinary nurses in the current study meta-regression result. This is in line with findings from Ethiopia (Wubante and Tegegne, 2022; Zewdie et al., 2023), China (Zhou et al., 2015; Zhao et al., 2022; Zhang et al., 2024), Ghana (Nkrumah et al., 2018), Sweden (Brämberg et al., 2017), Cyprus (Pitsillidou et al., 2020), Pakistan (Aslam et al., 2023), Switzerland (Verloo et al., 2017), the USA (Godshall, 2016; Stavor et al., 2017), Egypt (Baiomy and Khalek, 2015), and other studies (Azmoude et al., 2018; Bianchi et al., 2018; Fry and Attawet, 2018; Ayoubian et al., 2020; Chen et al., 2020; Clavijo-Chamorro et al., 2020; Duff et al., 2020; Ost et al., 2020; Spoon et al., 2020; Alqahtani et al., 2022; Clavijo-Chamorro et al., 2022; Crawford et al., 2022; Teixeira et al., 2022). This phenomenon could be attributed to the fact that head nurses, occupying leadership positions, tend to receive specialized training and orientation regarding the implementation of Evidence-Based Practice (EBP) and the adoption of new guidelines and procedures as provided by the Ministry of Health and regional health bureaus. Additionally, head nurses are tasked with the vital roles of overseeing and mentoring patient care and outcomes, facilitating new staff orientation, engaging in strategic planning, and dedicating sufficient time to conduct research. Furthermore, head nurses often engage in advocacy efforts for EBP during various conferences and meetings, thereby contributing to the dissemination and adoption of best practices within the healthcare community. This suggests that reinforcing the mentorship role of head nurses in promoting EBP within hospital settings will effectively enhance the adoption of EBP among nurses, fostering a culture where evidence-based approaches are embraced and integrated seamlessly into practice.

Nurses exhibiting effective communication skills were nearly five times more likely to implement EBP in their workplace compared to their counterparts, according to the results of the meta-regression analysis in this study. This finding is consistent with research conducted worldwide (Godshall, 2016; Stavor et al., 2017; Azmoude et al., 2018; van der Goot et al., 2018; Yiridomoh et al., 2020; Alshammari et al., 2021; Alqahtani et al., 2022; Clavijo-Chamorro et al., 2022; Crawford et al., 2022; Lanssens et al., 2022; Aslam et al., 2023). This correlation could be attributed to the fact that nurses who possess a favorable attitude towards EBP are inherently motivated to engage in various aspects of evidence-based care, such as contributing to positive patient outcomes, actively participating in research endeavors, and adhering to new clinical guidelines. In order to carry out these activities effectively, they naturally communicate smoothly with patients, families, nurse leaders, mentors, and organizers of research conferences. Therefore, enhancing the effective communication skills of nurses through various training and mentorship approaches is crucial for fostering the implementation of EBP.

Nurses who have access to EBP guidelines within their healthcare settings are nearly two times more likely to implement EBP compared to their counterparts, as indicated by the meta-regression results of this study. This finding is consistent with other studies (Lizarondo et al., 2019; Shayan et al., 2019; Alqahtani et al., 2020; Clavijo-Chamorro et al., 2022; Lanssens et al., 2022). This could be attributed to the necessity of providing nurses with comprehensive and up-to-date collections of resources tailored to the specific nature of their clinical practice environments. Access to relevant scientific databases also plays a crucial role in enabling nurses to locate current, high-quality research findings. Moreover, the availability of modern materials and equipment, including clinical consumables, diagnostic tools, and therapeutic aids, is essential for the effective implementation of guidelines and recent evidence within specific care contexts. Therefore, ensuring access to these resources can significantly enhance the implementation of EBP among nurses.

### Limitations

Articles published in languages other than English were omitted from the analysis. In mixed-method studies, risk bias assessment was limited to the quantitative component. Furthermore, no studies pertaining to the perspectives of patients or their families were identified, indicating that the review predominantly represents findings from the standpoint of healthcare providers. Despite encountering considerable heterogeneity, the data was pooled for analysis.

## Conclusion and recommendation

Only half of the nurses in Ethiopia demonstrate a robust uptake of EBP within clinical settings, which falls below the expectation of the Kampala Declaration, indicating a pressing need for concerted efforts to instill this valuable habit. This study underscores the pivotal role of several factors in facilitating EBP uptake among nurses, including a sound understanding of EBP principles, a positive attitude toward its application, proficient communication skills, leadership roles, and ready access to updated EBP guidelines. Consequently, it is imperative to prioritize capacity-building initiatives, such as targeted training programs, widespread dissemination of the latest EBP guidelines, and the bolstering of mentorship roles for head nurses. Additionally, fostering effective communication skills among nurses is paramount to promoting the uptake of EBP practices.

## Data Availability

The original contributions presented in the study are included in the article/Supplementary Material, further inquiries can be directed to the corresponding author.

## References

[B1] Alemayehu A. Jevoor P. C. (2021). Utilisation of evidence-based practice and its associated factors among nurses. Indian J. Continuing Nurs. Educ. 22 (2), 180–187. 10.4103/ijcn.ijcn_101_20

[B2] Alene Z. Bogale H. Fikadu A. Workina A. (2021). Assessment of evidence-based practice knowledge, utilization of practice and associated factors on nurses working in the adult intensive care unit of federal public. Hosp. Addis Ababa, 1–18. 10.21203/rs.3.rs-966691/v1

[B3] Alqahtani N. Oh K. M. Kitsantas P. Rodan M. (2020). Nurses’ evidence-based practice knowledge, attitudes and implementation: a cross-sectional study. J. Clin. Nurs. 29 (1–2), 274–283. PMID: 31714647. 10.1111/jocn.15097 31714647

[B4] Alqahtani N. Oh K. M. Kitsantas P. Rodan M. Innab A. Asiri S. (2022). Organizational factors associated with evidence-based practice knowledge, attitudes, and implementation among nurses in Saudi Arabia. Int. J. Environ. Res. Public. Health 19, 8407. 10.3390/ijerph19148407 35886258 PMC9324115

[B5] Alshammari M. S. Alshurtan R. Alsuliman G. Alshammari M. Alhamazani H. Alshammry S. (2021). Factors affecting the implementation and barriers to evidence-based practice among nurse practitioners in hail region, Saudi Arabia. Nurse Media J. Nurs. 11, 187–196. 10.14710/nmjn.v11i2.38329

[B6] Aslam S. Farooq Z. Azam S. Naheed F. Yaqoob R. (2023). Barriers in implementing evidence-based practice among nurses at tertiary care hospitals of lahore: barriers in implementing evidence-based practice. NURSEARCHER J. Nurs. Midwifery Sci. 31, 43–46. 10.54393/nrs.v3i02.53

[B7] Assefa K. Shewangizaw Z. (2021). Evidence-based practice utilization and associated factors among nurses in public hospitals, Addis Ababa, Ethiopia. Res. Square. 10.21203/rs.3.rs-477800/v1 PMC12044898 40307799

[B8] Aynalem Z. B. Yazew K. G. Gebrie M. H. (2021). Evidence-based practice utilization and associated factors among nurses working in Amhara Region Referral Hospitals, Ethiopia. PloS one 16 (3), 02488344–e248915. 10.1371/journal.pone.0248834 PMC7978364 33740000

[B9] Ayoubian A. Nasiripour A. A. Tabibi S. J. Bahadori M. (2020). Evaluation of facilitators and barriers to implementing evidence-based practice in the health services: a systematic review. Galen. Med. J. 9, e1645. 10.31661/gmj.v9i0.1645 34466560 PMC8343503

[B10] Azmoude E. Aradmehr M. Dehghani F. (2018). Midwives’ attitude and barriers of evidence based practice in maternity care. Malays. J. Med. Sci. 25, 120–128. 10.21315/mjms2018.25.3.12 30899193 PMC6422555

[B11] Baiomy S. Khalek E. A. (2015). Factors influencing effective implementation of evidence-based practice among nurses in Assiut City hospitals, Egypt: a comparative study. IOSR J. Nurs. Heal Sci. 4, 11–19. 10.9790/1959-04521119

[B12] Bianchi M. Bagnasco A. Bressan V. Barisone M. Timmins F. Rossi S. (2018). A review of the role of nurse leadership in promoting and sustaining evidence-based practice. J. Nurs. Manag. 26, 918–932. 10.1111/jonm.12638 30198088

[B13] Brämberg E. B. Nyman T. Kwak L. Alipour A. Bergström G. Schäfer Elinder L. (2017). Development of evidence-based practice in occupational health services in Sweden: a 3-year follow-up of attitudes, barriers and facilitators. Int. Arch. Occup. health Serv. 90 (4), 335–348. 10.1007/s00420-017-1200-8 PMC5387008 28204870

[B14] Branham S. DelloSritto R. Hilliard T. (2014). Lost in translation: the acute care nurse practitioners’ use of evidence-based practice: a qualitative study. J. Nurs. Educ. Pract. 4 (6), 53. 10.5430/jnep.v4n6p53

[B15] Burman M. E. Robinson B. Hart A. M. (2013). Linking evidence-based nursing practice and patient-centered care through patient preferences. Nurs. Adm. Q. 37 (3), 231–241. 10.1097/NAQ.0b013e318295ed6b 23744469

[B16] CEBHA (2012). Kigali declaration on evidence based healthcare in Africa. Kigali, Rwanda. Final version. Available at: http://cebha.org/sites/default/files/Kigali%20Declaration.pdf.

[B17] CEBHA (2014). Reflections on the CEBHA workshop on evidence based healthcare in Addis Ababa, Ethiopia. Available at: http://www.cebha.org/content/reflections-cebha-workshop-evidence-based-healthcare-addisAbabaEthiopia.

[B18] Chen L. L. Wu Y. N. Zhou C. L. Li X. X. Zhao H. H. (2020). Value, knowledge and implementation on evidence-based practice among nurse managers in China: a regional cross-sectional survey. J. Nurs. Manag. 28, 139–147. 10.1111/jonm.12907 31746069

[B19] Clavijo-Chamorro M. Z. Romero-Zarallo G. Gómez-Luque A. López-Espuela F. Sanz-Martos S. López-Medina I. M. (2022). Leadership as a facilitator of evidence implementation by nurse managers: a metasynthesis. West. J. Nurs. Res. 44, 567–581. 10.1177/01939459211004905 33853443

[B20] Clavijo-Chamorro M. Z. Sanz-Martos S. Gómez-Luque A. Romero-Zarallo G. López-Medina I. M. (2020). Context as a facilitator of the implementation of evidence-based nursing: a meta-synthesis. J. Nurs. Res. 43, 60–72. 10.1177/0193945920914397 32321372

[B21] Crawford C. L. Rondinelli J. Zuniga S. Valdez R. M. Tze-Polo L. Titler M. G. (2022). Barriers and facilitators influencing EBP readiness: building organizational and nurse capacity. Worldviews Evid. -Based Nurs. 20, 27–36. 10.1111/wvn.12618 36464805

[B22] Dagne A. H. Beshah M. H. Kassa B. G. Dagnaw E. H. (2021). Implementation of evidence-based practice and associated factors among nurses and midwives working in Amhara Region government hospitals: a cross-sectional study. Reprod. health 18, 36–40. 10.1186/s12978-021-01096-w 33579309 PMC7881559

[B23] Degu A. B. Yilma T. M. Beshir M. A. Inthiran A. (2022). Evidence-based practice and its associated factors among point-of-care nurses working at the teaching and specialized hospitals of Northwest Ethiopia: a concurrent study. PloS one 17 (5), 02673477–e267420. 10.1371/journal.pone.0267347 PMC9070954 35511810

[B24] Dereje B. Hailu E. Beharu M. J. (2019). Evidence-based practice utilization and associated factors among nurses working in public hospitals of jimma zone Southwest Ethiopia: a cross sectional study. Adv. Pharmacoepidemiol. Drug Saf. 7. 10.35248/2327-5146.7.321

[B25] Duff J. Cullen L. Hanrahan K. Steelman V. (2020). Determinants of an evidence-based practice environment: an interpretive description. Implement. Sci. Commun. 1, 85. 10.1186/s43058-020-00070-0 33043300 PMC7542098

[B26] Fry M. Attawet J. (2018). Nursing and midwifery use, perceptions and barriers to evidence-based practice: a cross-sectional survey. Int. J. Evid. -Based Healthc. 16, 47–54. 10.1097/XEB.0000000000000117 28759503

[B27] Furtado L. Coelho F. Mendonça N. Soares H. Gomes L. Sousa J. P. (2024). Exploring professional practice environments and organisational context factors affecting nurses' adoption of evidence-based practice: a scoping review. Healthcare 12 (2), 245. MDPI. 10.3390/healthcare12020245 38255132 PMC10815808

[B28] Godshall M. (2016). Fast facts for evidence-based practice in nursing: implementing EBP in a nutshell. 2nd edition. New York (NY): Springer Publishing Company.

[B29] Hadgu G. Almaz S. Tsehay S. (2015). Assessment of nurses’ perceptions and barriers on evidence based practice in tikur anbessa specialized hospital Addis Ababa Ethiopia. Am. J. Nurs. Sci. 4 (3), 73–83. 10.11648/j.ajns.20150403.15

[B30] Hoyiso D. Arega A. Markos T. (2018). Evidence based nursing practice and associated factors among nurses working in Jimma zone public hospitals, Southwest Ethiopia. Int. J. Nurs. Midwifery 10 (5), 47–53. 10.5897/IJNM2017.0294

[B31] Jane Vortherms M. Brenda Spoden B. Jill Wilcken B. (2015). From evidence to practice: developing an outpatient acuity-based staffing model. Clin. J. Oncol. Nurs. 19 (3), 332–337. PMID: 26000583. 10.1188/15.CJON.332-337 26000583

[B32] Jordan P. Bowers C. Morton D. (2016). Barriers to implementing evidence-based practice in a private intensive care unit in the Eastern Cape. South. Afr. J. Crit. Care 32 (2), 50–54. 10.7196/sajcc.2016.v32i2.253

[B33] Kim S. C. Stichler J. F. Ecoff L. Brown C. E. Gallo A.-M. Davidson J. E. (2016). Predictors of evidence-based practice implementation, job satisfaction, and group cohesion among regional fellowship program participants. Worldviews Evid. Based Nurs. 13 (5), 340–348. 10.1111/wvn.12171 27447125

[B34] Kristensen N. Nymann C. Konradsen H. (2015). Implementing research results in clinical practice: the experiences of healthcare professionals in Denmark. BMC Health Serv. Res. 16 (1), 48. 10.1186/s12913-016-1292-y PMC4748469 26860594

[B35] Labrague L. J. McEnroe-Pettite D. Tsaras K. D’Souza M. S. Fronda D. C. Mirafuentes E. C. (2019). Predictors of evidence-based practice knowledge, skills, and attitudes among nursing students. Nurs. Forum 54 (2), 238–245. 10.1111/nuf.12323 30582630

[B36] Lamesa D. Seifu W. Abdella J. Ezo E. (2023). Utilization of evidence-based nursing practice and associated factors among nurses working in saint Paul’s hospital millennium medical college, Ethiopia. SAGE Open Nurs. 9, 23779608231215599–8. 10.1177/23779608231215599 38020318 PMC10666712

[B37] Lanssens D. Goemaes R. Vrielinck C. Tency I. (2022). Knowledge, attitudes and use of evidence-based practice among midwives in Belgium: a cross-sectional survey. Eur. J. Midwifery 6, 36. 10.18332/ejm/147478 35794875 PMC9186072

[B38] Leen B. Bell M. McQuillan P. (2014). Evidence-based practice: a practice manual. ISBN: 9781908972033.

[B39] Li S. Cao M. Zhu X. (2019). Evidence-based practice: knowledge, attitudes, implementation, facilitators, and barriers among community nurses—systematic review. Medicine 98 (39), e17209–9. 10.1097/MD.0000000000017209 31574830 PMC6775415

[B40] Lizarondo L. Lockwood C. McArthur A. (2019). Barriers and facilitators to implementing evidence in african health care: a content analysis with implications for action. Worldviews Evid.-Based Nurs. 16, 131–141. 10.1111/wvn.12355 30977592

[B41] Megersa Y. Dechasa A. Shibru A. Mideksa L. Tura M. R. (2023). Evidence-based practice utilisation and its associated factors among nurses working at public hospitals in West Shoa zone, central Ethiopia: a cross-sectional study. BMJ open 13 (1), e063651–10. 10.1136/bmjopen-2022-063651 PMC9884937 36707114

[B42] Melnyk B. M. Fineout-Overholt E. (2022). Evidence-based practice in nursing & healthcare: a guide to best practice. Lippincott Williams & Wilkins.

[B43] Melnyk B. M. Gallagher-Ford L. Zellefrow C. Tucker S. Thomas B. Sinnott L. T. (2018). The first U.S. study on nurses’ evidence-based practice competencies indicates major deficits that threaten healthcare quality, safety, and patient outcomes. Worldviews Evid. Based Nurs. 15, 16–25. 10.1111/wvn.12269 29278664

[B44] Nkrumah I. Atuhaire C. Priebe G. Cumber S. N. (2018). Barriers for nurses’ participation in and utilisation of clinical research in three hospitals within the Kumasi Metropolis, Ghana. Pan Afr. Med. J. 30 (1), 1–11. ISSN 1937-8688. 10.11604/pamj.2018.30.24.15230 PMC6133193 30214657

[B45] Oluwatoyin F. E. EkeFJH B. F. (2016). Knowledge and utilization of evidence-based nursing practice among nurses of Offa Specialist Hospital, Kwara State. IOSR J. Nurs. Health Sci. 4, 51–62.

[B46] Ost K. Blalock C. Fagan M. Sweeney K. M. Miller-Hoover S. R. (2020). Aligning organizational culture and infrastructure to support evidence-based practice. Crit. Care Nurse 40, 59–63. 10.4037/ccn2020963 32476025

[B47] Page M. J. McKenzie J. E. Bossuyt P. M. Boutron I. Hoffmann T. C. Mulrow C. D. (2021). The PRISMA 2020 statement: an updated guideline for reporting systematic reviews. BMJ 372, n71. 10.1136/bmj.n71 33782057 PMC8005924

[B48] Pitsillidou M. Roupa Z. Farmakas A. Noula M. (2020). Barriers to the adoption of evidence-based practice among nurses. KONTAKT-Journal Nurs. Soc. Sci. Relat. Health & Illn. 22 (2), 85–91. 10.32725/kont.2020.017

[B49] Promoting evidence-based health care in Africa (2017). Promoting evidence-based health care in Africa. Bull. World Health Organ 95, 616–7. 10.2471/BLT.17.030917 28867841 PMC5578384

[B50] Riahi S. Khajehei M. (2019). Palliative care: a systematic review of evidence-based interventions. Crit. care Nurs. Q. 42 (3), 315–28. 10.1097/cnq.0000000000000269 31135482

[B51] Saleh U. S. (2018). Theory guided practice in nursing. J. Nurs. Res. Pract. 2 (1).

[B52] Shayan S. J. Kiwanuka F. Nakaye Z. (2019). Barriers associated with evidence‐based practice among nurses in low‐and middle‐income countries: a systematic review. Worldviews Evidence‐Based Nurs. 16 (1), 12–20. 10.1111/wvn.12337 30604471

[B53] Spoon D. Rietbergen T. Huis A. Heinen M. van Dijk M. van Bodegom-Vos L. (2020). Implementation strategies used to implement nursing guidelines in daily practice: a systematic review. Int. J. Nurs. Stud. 111, 103748. 10.1016/j.ijnurstu.2020.103748 32961463

[B54] Stavor D. C. Zedreck-Gonzalez J. Hoffmann R. L. (2017). Improving the use of evidence-based practice and research utilization through the identification of barriers to implementation in a critical access hospital. J. Nurs. Adm. 47 (1), 56–61. 10.1097/NNA.0000000000000437 27926624

[B55] Stokke K. Olsen N. R. Espehaug B. Nortvedt M. W. (2014). Evidence based practice beliefs and implementation among nurses: a cross-sectional study. BMC Nurs. 13 (1), 8–10. 10.1186/1472-6955-13-8 24661602 PMC3987836

[B56] Tadesse B. Teshome H. W. Eyoel A. D. (2018). Assessment of nurses knowledge and utilization of evidence based practice and its associated factors in selected hospitals of southern Ethiopia. Adv. Pract. Nurs. 3, 150. 10.4172/2573-0347.1000150

[B57] Teixeira A. C. Nogueira A. Barbieri-Figueiredo M. D. (2022). Professional empowerment and evidence-based nursing: a mixed-method systematic review. J. Clin. Nurs. 32, 3046–3057. 10.1111/jocn.16507 36039039

[B58] van der Goot W. E. Keers J. C. Kuipers R. Nieweg R. M. B. de Groot M. (2018). The effect of a multifaceted evidence-based practice programme for nurses on knowledge, skills, attitudes, and perceived barriers: a cohort study. Nurse Educ. Today 63, 6–11. 10.1016/j.nedt.2018.01.008 29407262

[B59] Verloo H. Desmedt M. Morin D. (2017). Beliefs and implementation of evidence‐based practice among nurses and allied healthcare providers in the Valais hospital, Switzerland. J. Eval. Clin. Pract. 23 (1), 139–48. 10.1111/jep.12653 27687154

[B60] WHO (2015). Nurses and midwives: a vital resource for health. European compendium of good practices in nursing and midwifery towards Health 2020 goals. Copenhagen: WHO Regional Office for Europe. Available at: http://www.euro.who.int/en/healthtopics/Health-systems/nursing-and-midwifery/publications/2015/nurses-and-midwives-a-vital-resource-for-health.-european compendium-of-good-practices-in-nursing-and-midwifery-towards-health-2020-goals (Accessed July 2, 2017).

[B61] Williams B. Perillo S. Brown T. (2015). What are the factors of organizational culture in health care settings that act as barriers to the implementation of evidence-based practice? A scoping review. Nurse Educ. today 35 (2), e34–e41. PMID: 25482849. 10.1016/j.nedt.2014.11.012 25482849

[B62] World health organization (WHO) (2017). Facilitating evidence-based practice in nursing and midwifery in the WHO European region. United Kingdom: WHO, 9–10.

[B63] Wubante S. M. Tegegne M. D. (2022). Evidence-based practice and its associated factors among health professionals in Ethiopia: systematic review and meta-analysis. Inf. Med. Unlocked 32, 101012. 10.1016/j.imu.2022.101012

[B64] Yiridomoh G. Y. Dayour F. Bonye S. Z. (2020). Evidence-based practice and rural health service delivery: knowledge and barriers to adoption among clinical nurses in Ghana. Rural. Soc. 29, 134–149. 10.1080/10371656.2020.1795350

[B65] Zewdie A. Ayele M. Melis T. Kasahun A. W. (2023). Determinants of evidence-based practice among health care professionals in Ethiopia: a systematic review and meta-analysis. Plos one 18 (11), e0293902. 10.1371/journal.pone.0293902 37943797 PMC10635493

[B66] Zhang Y. Guo Z. Xu S. Yao M. Feng X. Lan M. (2024). Facilitating evidence-based practice among nurses in a tertiary general hospital: a six-year practice of an implementation strategy informed by the i-PARIHS framework. J. Nurs. Manag. 19, 1–7. 10.1155/2024/8855667 PMC11919120 40224874

[B67] Zhao J. Bai W. Zhang Q. Su Y. Wang J. Du X. (2022). Evidence-based practice implementation in healthcare in China: a living scoping review. Lancet Regional Health–Western Pac. 20, 100355. 10.1016/j.lanwpc.2021.100355 PMC8743207 35036975

[B68] Zhou F. Maier M. Hao Y. Tang L. Guo H. Liu H. (2015). Barriers to research utilization among registered nurses in traditional Chinese medicine hospitals: a cross-sectional survey in China. Evid. Based Complementary Altern. Med. 2015, 475340–8. 10.1155/2015/475340 PMC4663293 26649060

