# Supplementary material for: Uptake of evidence-based practice and its predictors among nurses in Ethiopia: a systematic review and meta-analysis
Source: Front Pharmacol. 2024 Jul 18;15:1421690. doi: 10.3389/fphar.2024.1421690 (PMC11291372; doi:10.3389/fphar.2024.1421690)
Supplement: Supplementary file 2 [file Table2.pdf]

Supplementary file 2: Search strategy used to retrieve eligible studies.

S. No	Data base	Searching Combination	# article (Total record)	Searching date
3	PubMed	((((((((Evidence-based practice) AND (implementation)) OR (uptake)) OR (utilization)) AND (associated factors)) OR (predictors)) OR (determinants)) AND (among nurse*)) AND (in Ethiopia)	520	February 12, 2024
4	Scopus	TITLE-ABS-KEY (Evidence-based practice) AND TITLE-ABS-KEY ("implementation* " OR " uptake " OR utilization ") AND TITLE-ABS-KEY ("associated factors" OR " predictors " OR determinants ") AND TITLE-ABS-KEY ("among nurse*") AND TITLE-ABS-KEY ("in Ethiopia")	367	February 7, 2024
5	EMBASE	(Evidence-based practice) AND (implementation)) OR (uptake)) OR (utilization)) AND (associated factors)) OR (predictors)) OR (determinants)) AND (among nurse*)) AND (in Ethiopia)	313	February 12, 2024
**Subtotal from databases**			**1200**	
9	Google scholar	Uptake of Evidence-based practice and its predictors among nurses in Ethiopia	289	February 7, 2024
11	Wiley Online Library	Uptake of Evidence-based practice and its predictors among nurses in Ethiopia	89	February 7, 2024
13	University repositories	Uptake of Evidence-based practice and its predictors among nurses in Ethiopia*	12	February 12, 2024
14	Across references		12	
**Subtotal from other sources**			**390**	
**Total number of retrieved articles**			**2590**	
